# Multifaceted Imaging of Renal Lesions With an Emphasis on Cross-Sectional Imaging

**DOI:** 10.7759/cureus.59956

**Published:** 2024-05-09

**Authors:** Rahul Dev, Udit Chauhan, Khanak K Nandolia

**Affiliations:** 1 Diagnostic and Intervention Radiology, All India Institute of Medical Sciences, Rishikesh, IND

**Keywords:** renal cell carcinoma, computed tomography (ct) imaging, magnetic resonance imaging (mri), bosniak classification, renal lesions

## Abstract

Introduction

Renal lesions are common findings encountered in cross-sectional imaging. Ultrasonography (USG), computed tomography (CT), and magnetic resonance imaging (MRI) are available modalities for evaluating renal lesions. The Bosniak classification system aids in classifying a renal lesion into a particular category based on various imaging characteristics on contrast-enhanced CT (CECT).

Materials and methods

The CT report archives were searched for the keyword 'Bosniak' lesions, and six illustrative cases were selected to be included in the review.

Results

Six cases under Bosniak categories I to IV were included in the review. Operative follow-ups were added in cases where patients underwent surgery.

Discussion

We have reviewed the imaging features of various renal lesions with cross-sectional modalities, namely CT and MRI, with special emphasis on the Bosniak classification system, including its amendments.

Conclusion

The Bosniak system is widely used to classify and characterize renal lesions. The authors have presented a scoping review of the features of renal lesions and the Bosniak system.

## Introduction

Even after more than 30 years of Bosniak classification, renal lesions continue to puzzle radiologists [1]. Renal lesions can be detected by ultrasonography (USG), computed tomography (CT), or magnetic resonance imaging (MRI). Many lesions are asymptomatic and detected incidentally. Some lesions can present with alarming symptoms like hematuria, abdominal pain, or constitutional symptoms like weight loss, fever, or weakness. The pathological spectrum ranges from simple cortical cysts to complex neoplasms. Lesions can be congenital, infective, inflammatory, or neoplastic. Neoplasms are further divided depending on histopathological grade and cell type. Due to the wide pathological spectrum of renal lesions, it is important to differentiate benign 'touch me not' lesions from aggressive lesions requiring definitive management. The definite diagnosis depends on histopathology. Hence, the aim of imaging is to classify a lesion according to imaging features and guide the clinician in appropriate management [2].

## Materials and methods

This retrospective study was performed in the Department of Diagnostic and Intervention Radiology of the All India Institute of Medical Sciences, Rishikesh, India, after obtaining permission from the hospital medical records division. The cross-sectional CT imaging reports archive of the previous two years (January 2022 to January 2024) was searched for the keyword 'Bosniak category’ to look for reports containing various Bosniak category renal lesions. Images of selected patients were retrieved from the archives. Patients with a history of renal trauma, prior renal surgery, drainage of renal abscesses, or collection were excluded. Operated cases of renal malignancies were excluded. After applying the exclusion criteria, 512 reports were found. Clinical details of the patients were fetched from the hospital information system. The histopathology of the lesions was reviewed in cases where the patients were operated on. Six illustrative cases were selected for the article. Scans were performed after obtaining written, informed consent from the patients.

Intravenous iodinated non-ionic water-soluble contrast iohexol (350 iodine/ml) was used with a wide-bore (18-20 gauge) venous cannula. Contrast injection was performed at a rate of 3-4 ml/second with a dose of 2 ml/kg body weight using a pressure injector with 20 ml of saline bolus. None of the patients had developed adverse contrast reactions. Scans were acquired on a Philips Brilliance 64-slice CT scanner (Philips, Amsterdam, Netherlands). Non-contrast images were first acquired, followed by corticomedullary, nephrographic, and excretory phases. A slice thickness of 5 mm was used in the acquisition with thin reconstruction with 0.5 mm slice thickness in volumetric data. Images were analyzed on Philips IntelliSpace (Philips, Amsterdam, Netherlands) workstations by radiologists with more than 10 years of experience in cross-sectional imaging. Lesions were evaluated in three orthogonal planes. Non-contrast CT images were evaluated for baseline Hounsfield unit (HU) values, calcifications, fat components, and the presence of bleeds. Post-contrast images were evaluated for enhancing solid components, septations, and internal architecture. Some lesions showed extrarenal extension into the retroperitoneum and perinephric space.

## Results

The CT images of six patients were selected as illustrative cases to demonstrate the unique imaging features of Bosniak categories I, II, IIF, III, and IV. Details of the six patients are provided in Table 1. Images were analyzed on workstations, and illustrative images were selected.

**Table 1:** Demographics and clinical details of the illustrative cases included in our study CECT: contrast-enhanced CT

Case	Age and gender	Clinical history	CT imaging findings	Follow-up
1	30 years old, male	The patient is a known case of autosomal dominant polycystic kidney disease, presenting with recurrent bilateral lumbar pain.	Multiple Bosniak I and II cysts in both kidneys	The patient was managed conservatively. Serial imaging was recommended due to the syndromic status of the patient.
2	40 years old, female	The patient underwent CECT of the abdomen with a pancreatic protocol for evaluation of recurrent abdominal pain and elevated serum lipase.	The patient was incidentally diagnosed with a Bosniak II cyst in her left kidney.	The patient was managed conservatively.
3	50 years old, male	The patient presented with dull, aching pain in the right lumbar region for six months. A cyst with a complex structure was detected on USG. A multiphasic CECT was done for the evaluation of the renal lesion.	A Bosniak IIF cyst was detected in the right kidney.	The patient was managed conservatively. Imaging follow-up was recommended on CECT.
4	20 years, male	The patient had a known case of tuberous sclerosis and a recurrent refractory seizure. Screening USG of the abdomen showed multiple hyperechoic lesions in both kidneys. A multiphasic CECT abdomen was done for the characterization of renal lesions.	Bosniak III lesions with fat contents in both kidneys—angiomyolipoma	The patient was managed conservatively. Serial imaging was recommended due to the syndromic status of the patient.
5	60 years, male	The patient underwent an ultrasound of the kidneys for lumbar pain and a single episode of hematuria. Ultrasound showed an exophytic cyst in the right kidney. A CECT was done to characterize the lesion.	Bosniak III lesion in the right kidney	The patient underwent a partial nephrectomy. A papillary variant of renal cell carcinoma was detected on histopathology.
6	65 years, male	The patient presented with recurrent hematuria, abdominal pain, and weight loss. Ultrasound showed solid cystic lesions with vascularity in the solid component of the lesions. The CECT demonstrated enhancement in the solid component and the central necrotic component.	Bosniak IV lesions in both kidneys	A biopsy of the lesions proved the diagnosis of an aggressive clear-cell variant of renal cell carcinoma.

## Discussion

Technical considerations: USG, CT, and MRI

Ultrasonography

A USG is the first line of investigation for evaluating renal lesions. It is commonly available, less costly, devoid of ionizing radiation, and has no absolute procedural contraindications. The renal lesions can be labelled as simple or complex on USG. A renal lesion is labelled as a simple cyst on USG when it has well-defined margins, anechoic fluid contents, post-acoustic enhancement, and an absence of internal vascularity [1, 2]. Lesions that do not fit above are labelled complex lesions and can be further characterized by CT or MRI, especially in the Bosniak classification. The major limitation of USG is its operator dependency [3, 4]. Newer techniques, like contrast-enhanced USG (CEUSG), are promising for indeterminate lesions. Densely calcified lesions are obscured, and their internal contents cannot be assessed due to posterior echo. Inherent limitations of USG are suboptimal assessment of deep-seated lesions, hindrance by adjacent bowel gases, and patient physical habitus. Multiphasic contrast-enhanced CT (CECT) is the imaging modality for evaluating complex renal lesions because of its wide availability and better spatial and temporal resolution. Enhancement on CT depends upon the cellularity and vascularity of the lesion, intravenous contrast being administered, its dose, and injection rates [1].

Computed Tomography

Non-contrast-enhanced CT (NCCT) images are acquired to see baseline lesion attenuation, hyperdense hemorrhagic/proteinaceous contents, fatty contents, calcifications, and incidental urolithiasis. The post-contrast corticomedullary phase is acquired to assess vasculature, including tumoral neovascularity and renal vessel anatomy. The nephrographic phase optimizes the detection of intrarenal lesions and delineates pseudo-lesions of the corticomedullary phase and hypo-enhancing tumours. The delayed or excretory phase can evaluate the anatomy of the collecting system and ureters, the involvement of calyces, pelvis, or ureters, and excretory renal function [1, 5-9]. Lesions are evaluated on CECT for features like absolute and dynamic enhancement and the presence of internal septa or solid components. Non-contrast-enhanced CT and CECT images are compared for hyperdense contents and spurious enhancement [2, 5, 6]. Multiphasic CECT acquisition is recommended since various tumours show unique kinetics for contrast enhancement, such as early arterial enhancement in clear cell renal cell carcinoma (RCC) and delayed enhancement in the papillary RCC [2]. Acquisition parameters, such as exposure factors, slice thickness, and slice overlap, are adjusted to optimize image quality. Acquisition timing is crucial for detecting small, enhancing lesions and solid nodules in cystic lesions. The thickness of the acquisition slice is usually 3-5 mm. Post-processing, such as thin slice reconstruction, multiplanar reconstruction (MPR), and 3D surface rendering, is performed. Thin-slice MPR is a must for detecting cystic masses and solid nodules [5]. An MRI can be an alternative to CECT in cases of iodinated contrast allergies. Contrast-enhanced MRI (CEMRI) is a multiphasic dynamic study that acquires corticomedullary, nephrographic, and excretory phase images. Other MR sequences, such as T1, T2, MR angiography, and urography, further help in lesion characterization and management [6].

Magnetic Resonance Imaging

Non-contrast MRI is radiation-free. Newer, faster sequences with parallel imaging can significantly reduce scanning time and require shorter breath-holding durations during acquisition. Due to the availability of newer macrocyclic MR contrast agents, the incidence of nephrogenic systemic fibrosis is lower. An MRI is a problem-solving tool, especially for indeterminate feature lesions on CT [2]. Enhancing septa or solid components increases the probability of the lesion being malignant and assigns a higher Bosniak category [3, 6, 7]. The presence of a solid component classifies the lesion in Bosniak category IV. Fat-saturated T1-weighted CEMRI offers better soft tissue contrast, so MRI can better evaluate indeterminate lesions [2]. Enhancement is quantified in MRI by calculating voxel signal intensity, similar to the HU values used in CT. A 15% increase in voxel signal intensity has 95% specificity to discriminate between benign and malignant lesions [1, 8]. The acquisition plan can be planned depending on the tumour's location. The sagittal plane is invariably acquired, with coronal and axial planes for polar and nonpolar lesions. This approach is most critical for patients with resectable lesions in the setting of a solitary kidney [6].

Overview of Bosniak classification

The Bosniak system is the most widely used and well-accepted imaging-based classification system for renal lesions, devised in 1984 by Dr. Morton Bosniak [7-14]. It was based on CECT initially and amended in 1997, 2005, and 2019. The 2019 version also included MRI features [7, 9]. The Bosniak system includes a broad spectrum of renal lesions, which are benign, intermediate, and malignant. The detection rate of renal lesions has increased due to increased life expectancy and the increased availability of imaging. The prevalence rate was 41% for renal lesions in the general population. This system is a starting point for evaluating and managing renal lesions. Lesions are categorized based on their benign or malignant nature for uniform communication between radiologists and treating surgeons [12]. The Bosniak system has categories I to IV. Due to their unambiguous and straightaway imaging features, category I and IV lesions are classified as benign or malignant, respectively. Decision-making is challenging with category II, IIF, and III lesions due to overlapping and ambiguous imaging features [12]. We will discuss lesions in each category individually, with illustrative cases. Illustrative cases are sourced from the teaching case files of the authors.

Bosniak Category I

**Figure 1:**
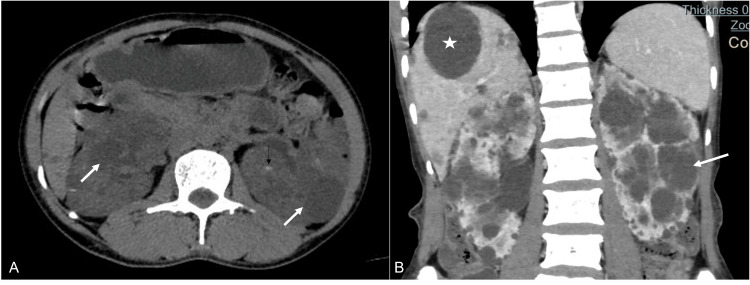
A young male patient with recurrent lumbar pain Axial NCCT (A) and coronal reformatted CECT (B) images show multiple hypodense cysts randomly distributed in both kidneys (short white arrows), which are classified as Bosniak I cysts. A few cysts show internal hyperdense fluid layering, suggesting internal haemorrhages (black arrow), which are categorized as Bosniak II cysts. The liver also shows multiple simple cysts (white asterisk). NCCT: non-contrast-enhanced CT; CECT: contrast-enhanced CT

Category I lesions (Figure 1) show homogenous water attenuation, imperceptible walls, and no enhancement and are primarily benign renal cortical cysts. Renal cortical cysts are by far the most common lesions detected in imaging [10]. Advancing age, male gender, smoking, and the presence of urinary tract obstruction are important risk factors for renal cyst development. Male patients have a higher mean number and higher mean diameter of cortical cysts [10, 11]. Septa and enhancement are absent in category I lesions. Complicated cystic lesions with hyperdense contents and <3cm diameter are also benign and managed conservatively. On MRI, the fluid shows a homogenous water signal [6]. All the category I lesions should be left alone and imaged only when symptomatic [15].

Bosniak Category II

**Figure 2:**
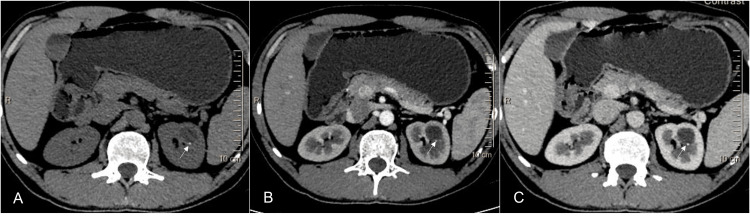
A 40-year-old female with an incidentally detected left renal cyst Axial NCCT (A), CECT corticomedullary phase (B), and nephrographic phase (C) images show a cyst with homogenous fluid density in the left kidney, with an eccentric speck of calcification along its posterior wall (white arrow). The cyst wall is imperceptible and non-enhancing. This represents a Bosniak II cyst. NCCT: non-contrast-enhanced CT; CECT: contrast-enhanced CT

Category II lesions (Figures 1, 2), although benign, raise concern due to the complexity of their internal architecture due to hyperdensity, septations, or infection. Hyperdensity is attributable to blood, dense contents, or soft calcium [2]. The majority of category II lesions are managed conservatively [9]. Some category II lesions may show one to three septa with non-linear morphology. Lesions with walls with minimal thickening (3 mm) and wall enhancement or multiple (>4 mm) smooth-enhancing thin (<2 mm) septa are classified in the IIF category. Here, F stands for follow-up. The MR of these lesions may show a hyperintense signal on T1 fat-saturated images. These lesions are monitored serially on imaging at six months, one year, and so on for at least five years, more frequently in elderly patients. Lesions may remain stable or progress into a higher-category lesion [1, 12].

Bosniak Category IIF

**Figure 3:**
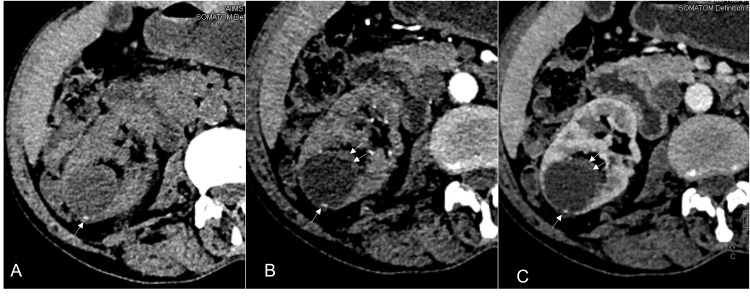
A 50-year-old male presented with right lumbar pain and a right renal cyst detected on ultrasound. Axial NCCT (A), CECT corticomedullary phase (B), and nephrographic phase (C) images show a cyst with homogenous water density in the right kidney with an eccentric calcification speck along its posterior wall (single arrow). The anterior cyst wall is minimally thickened (<3 mm) and shows enhancement (double arrows). This is categorized as a Bosniak IIF cyst. NCCT: non-contrast-enhanced CT; CECT: contrast-enhanced CT

Category IIF (Figure 3) was introduced and placed between II and III, based on surgical experience and the outcome of resecting category III lesions. These lesions may show thick, chunky calcifications and perceivable septal or wall enhancement, but no appreciable enhancement of the soft tissue component. Perceivable enhancement is believed to be due to tiny capillaries within the septa or walls [13]. The size criteria for IIF lesions were removed in the 2019 version of the classification as none of these lesions were found to be malignant [14]. Indeterminate category IIF/III lesions should be placed in category IV if the patient has features of syndromic involvement [1, 13, 14]. An MRI may be helpful in such cases as a problem-solving tool due to its ability to produce better soft tissue contrast. If an MRI examination cannot be undertaken, a percutaneous biopsy can be attempted, especially for the high-risk lesions [2].

For the management of IIF lesions, some consider follow-up a safe strategy, with follow-up every year for one to four years [14]. At the same time, other studies advocate resecting them in young patients, as no supporting data define a follow-up timeline [13]. An image-guided biopsy can come to the rescue to establish a definitive diagnosis of the lesion's solid or enhancing component [2]. Post-biopsy changes can alter a lesion's imaging appearance and make follow-up more challenging [6, 13]. Hwang et al., in their study on the follow-up of 201 patients with IIF lesions, found that 14 lesions showed radiological progression to category III over twenty months. Among these, 10 lesions were RCC, predominantly clear cell variants [16]. Hence, a biopsy cannot replace surgical exploration, as an indeterminate lesion can still harbour malignancy and will require surgical resection sooner or later. A study by Balyemez et al. retrospectively evaluated the database of lesions in the IIF or higher category and their MRI features. This study evaluated average diffusion coefficient (ADC) values using follow-up imaging or pathological diagnosis. The ADC values were significantly lower in malignant lesions than in benign ones. The ADC value cut-off of 2.28 x 10-6 mm2/s or less was 93% specific for labelling a lesion as malignant [17]. Hence, CEMRI with ADC values can be a problem-solving tool.

Bosniak Category III

**Figure 4:**
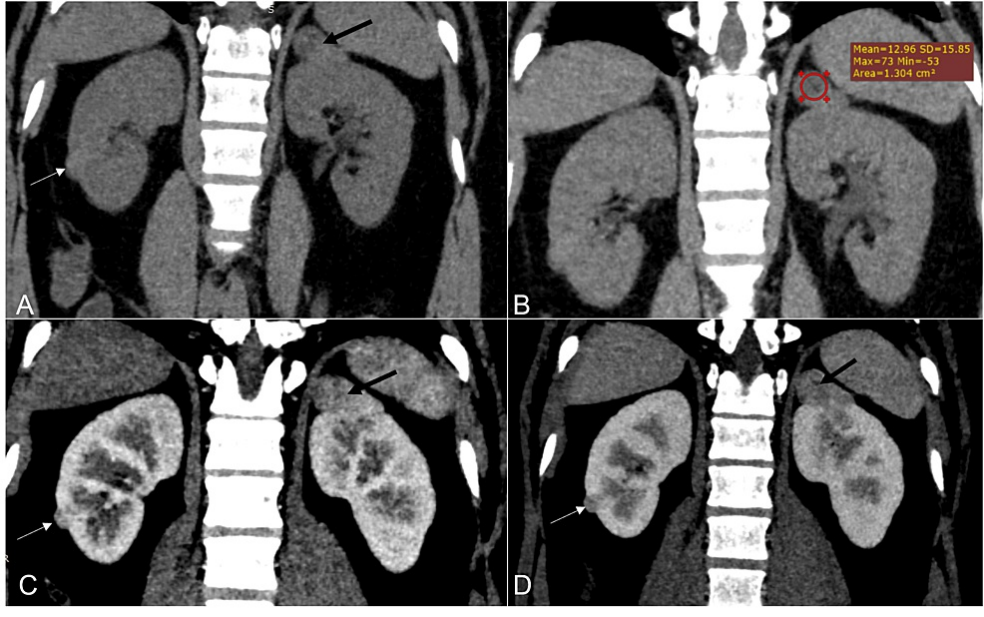
A 20-year-old male patient diagnosed with tuberous sclerosis Reformatted coronal NCCT (A & B), CECT corticomedullary phase (C), and nephrographic phase (D) images show multiple lesions in both kidneys (white and black arrows). The lesion in the left kidney upper pole shows fat attenuation with HU values of -53 HU in the ROI (B). Lesions in both kidneys show enhancement in the corticomedullary phase (C) and washout in the nephrographic phase (D), consistent with lipid-poor angiomyolipoma. This represents a Bosniak III lesion. NCCT: non-contrast-enhanced CT; CECT: contrast-enhanced CT; HU: Hounsfied unit; ROI: region of interest

Category III (Figure 4) includes a broad spectrum of lesions with a near-similar incidence of benign and malignant lesions. The spectrum includes unilocular and multilocular benign lesions, infected cysts, and malignant multilocular malignancies. All category III lesions show thick (>4 mm) measurable walls, septa, and solid components (<3 mm protrusion). Surgical resection of these lesions is preferred due to the higher incidence of malignancies in this category [15,16]. Follow-up is recommended for elderly patients and patients with renal dysfunction, as the loss of renal tissue may further worsen renal function. Follow-up imaging is performed six monthly for two years, followed by yearly [13, 14]. Nephrectomy carries a high mortality rate and a decreased five-year overall survival rate [11].

**Figure 5:**
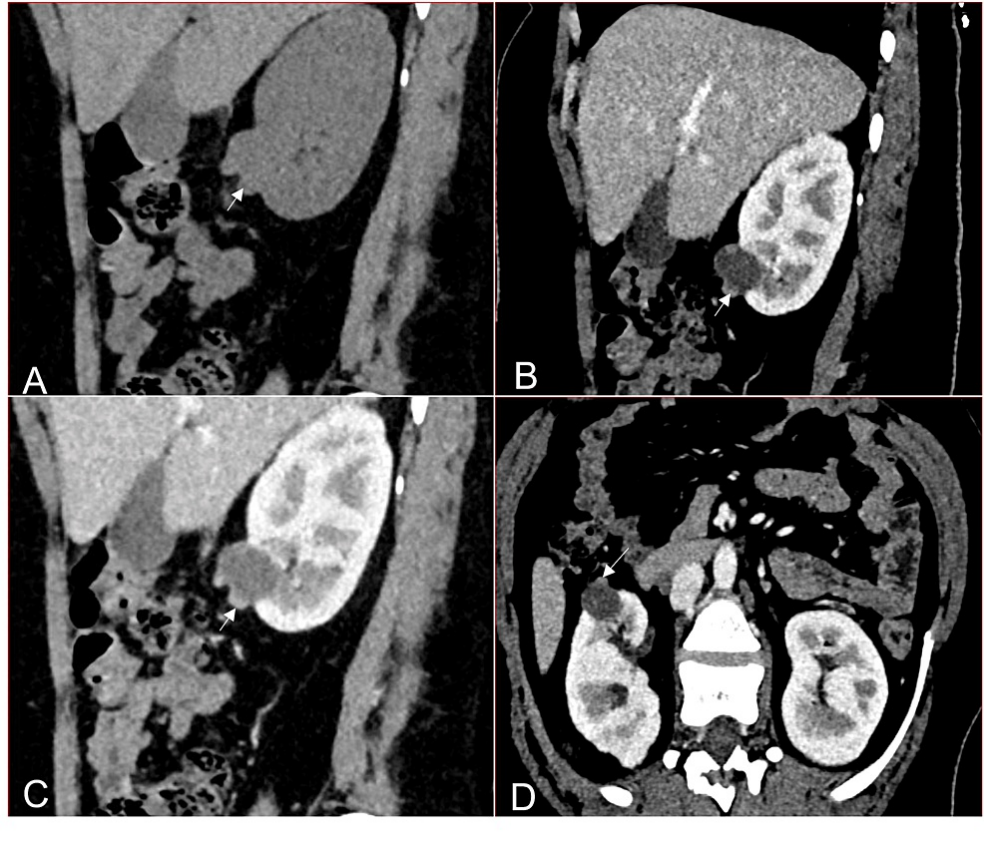
A 60-year-old male with a right renal lesion Reformatted oblique NCCT (A), corticomedullary phase (B), and nephrographic phase (C, D) images show an exophytic cyst in the right kidney lower pole. The cyst is hypodense with an eccentric enhancing nodule <3 mm in thickness (white arrows). This has been categorized as a Bosniak III lesion. The nodule shows enhancement in the corticomedullary phase and nephrographic phase. NCCT: non-contrast-enhanced CT

Category III lesions with clinical features of an infective or inflammatory nature require a follow-up CECT at four to eight weeks to guide further management [2, 13, 14, 18]. Increasing the follow-up interval in this subset of lesions gives a better category assignment as the lesion features evolve and become more evident with time progression [19, 20, 21, 22]. Herts et al. suggested continuing follow-up for at least five years [23]. Two studies by Patel et al. and Huang et al. recommended active surveillance in elderly patients, as overall cancer-free survival was the same as surgery outcomes with lower mortality and cardiovascular morbidity [19,20]. The vital role of radiologists is to convey to the surgeon the likelihood of the lesion under evaluation being either benign or malignant to guide further management. In the past, surgeons preferred to remove small and polar-located category III lesions [9]. Pruthi et al. tried to further define category III lesions by subdividing them into IIIn (cysts with wall nodularity) (Figure 5), and IIIs (cysts with enhancing septa). The IIIn lesions are more likely to progress to category IV on follow-up imaging. IIIs-IIF category lesions and IIIn-IV category lesions show similar imaging features. Recommended follow-up timelines for IIIs lesions are six and 12 months, biennially after that; IIIn lesions were recommended to be imaged at six and 12 months, annually after that [21].

Bosniak Category IV

**Figure 6:**
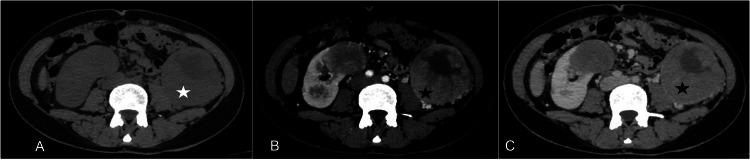
A 65-year-old male patient with recurrent hematuria, abdominal pain, and weight loss Axial NCCT (A), corticomedullary phase (B), and nephrographic phase (C) images show exophytic lesions arising from both kidneys. The lesions were hypodense (white asterisk) with an eccentric enhancing component >10 mm in thickness (black asterisk). This has been classified as Bosniak IV lesions. On histopathology, the lesions proved to be renal cell carcinoma. NCCT: non-contrast-enhanced CT

Category IV (Figure 6) lesions show measurable enhancement of the wall and septa, with distinct solid enhancing components [13]. These lesions show overlapping imaging features with category III lesions and require cross-sectional imaging, preferably CEMRI in all cases, followed by referral for surgical management [14-16, 18]. The solid nodular components in category IV lesions show a thickness of >4 mm or acute margins, while solid components in category III lesions show obtuse margins and <3 mm thickness.

Bosniak classification: 2019 update

The CEMRI features were added for each lesion category in addition to the CECT feature. A CEMRI is frequently seen to upstage the Bosniak category of a lesion over CECT because MRI shows better enhancement, internal architecture, and solid components [7]. In their study, Zhong et al. found that MRI upstaged lesions of various categories, namely II to IIF and IIF to III, due to better appreciation of septa, enhancement, and the presence of haemorrhage, and III to IV by detecting mural nodules [22].

The 2019 version of the Bosniak classification has added 'lesion types' under classes II and III. This new scheme tries to mitigate problems related to erroneous reporting, particularly concerning Bosniak III lesions, by objectively defining the thickness of septa and/or nodular protrusion [14]. This classification update recommends removing perceivable or measurable enhancement and replacing it with enhancement measured either qualitatively or quantitatively. Hypodense lesions covered under the umbrella term "too small to be characterized" are benign lesions [11, 14].

Solid components presenting as enhancing mural nodules are defined by objective, measurable parameters for both cross-sectional imaging modalities. The MRI-based Bosniak classification retained the number of septa, their morphology, and thickness parallel to the CT classification. The MRI also added signal intensity changes to the T1 and T2 sequences. They also recommended keeping the term 'cystic mass' for the IIF category onward lesion, mentioning the probability of malignancy and suggesting follow-up or management as these lesions can harbour papillary or clear cell variants of RCC.18. Those measuring <3 cm in size and lacking interval growth show a low risk of metastasis [11].

Other proposed inferences were removing size criteria for IIF lesions and attributing any form of calcification to being benign.

Imaging features

Calcifications: Friend or Foe

Calcification within a lesion is best appreciated on NCCT. A USG is susceptible to numerous artifacts. An MRI has low sensitivity for detecting calcifications within a lesion. Paradoxically, CECT lags in detecting enhancement in a calcified mass. At the same time, MRI is superior to CT in soft tissue contrast, especially for appreciating even a subtle enhancement within a calcified mass. Enhancement in a calcified lesion is designated as an ominous marker [1, 7]. Specks of calcifications, layered calcifications, and calcifications without associated enhancing soft tissue components are seen in benign lesions and can be left alone or followed up appropriately. Thick and nodular calcification and calcifications associated with wall enhancement or solid enhancing components are features of concern. These lesions must be placed in category III or IV and require active management [1, 13]. The morphology and distribution of calcifications can aid in classifying lesions into appropriate categories [9]. One of the studies advocated keeping all forms of calcification, suggesting a benign nature [14].

Lesion Density on CT

A lesion density is measured on the NCCT. If a lesion shows attenuation of -9 to 20 HU, it is hypodense, and attenuation of >70 HU is hyperdense. Hyperdensity in a renal lesion is attributable to blood products, proteinaceous contents, infection, and high-density neoplasms like cystic RCC. Lesions showing NCCT attenuation of -9 to 20 HU or >70 HU, porto-venous phase attenuation of <30 HU and homogenous enhancement are more likely to be benign. Bosniak suggested NCCT attenuation <20 HU for labeling a lesion as benign [9, 11]. Lesions with NCCT attenuation >20 HU, similar to adjacent renal parenchyma, may contain solid components. Such lesions require further evaluation with CEMRI or CECT [23]. If a contrast study is not feasible, USG can define the fluid nature of the cyst content to differentiate large cysts from cystic neoplasms [1, 9]. Hartman et al. highlighted one such case of cystic RCC masquerading as benign hyperdense cysts on CT [24]. Hyperdense cortical cysts are incidental and isolated findings. They are smaller and seen along the kidney periphery [9]. 

Lesion Signal on MRI

An MRI signal is defined on the T1 and T2 sequences as compared to a skeletal muscle signal. Similarly, the T2 signal similar to the water signal and the non-enhanced T1 signal >2.5 times to adjacent renal parenchyma were considered benign [14]. The hyperintense signal on T1 fat-saturated images denotes the proteinaceous or hemorrhagic nature of the fluid content. Such lesions require contrast administration and subtraction imaging [23].

Septations: Morphology and Enhancement

The Bosniak classification version 2019 defines a septum as a linear or curvilinear structure bridging two lesion surfaces [14]. Septations are considered features of the dynamic process within a renal lesion secondary to cyst growth, haemorrhages, calcifications, or the development of the internal solid components. Few studies defined the septal thickness threshold as 2 mm for thin septa and 4 mm for thick septa. This definition reduces false positive interpretations and avoids surgical interventions. These studies recommend using the uniform term 'thin septa' instead of various terms like 'hairline septa' or 'pencil thin septa' [13, 14]. To remove subjectivity, a cut-off of 4 mm was suggested for defining septal thickness, which increased the specificity of the categorizing lesion.

Thin and non-enhancing septa are seen in benign lesions, while thick and nodular septa, showing measurable enhancement, are indicators of the potential malignant nature of the lesion [1]. The irregular, thick walls and nodules help to differentiate category III lesions from IV lesions [14]. Malignant lesions with septations may have hemorrhagic fluid within, which may hinder the assessment of enhancement on CECT. A CEMRI can be useful in such cases as a problem-solving tool [23].

The Bosniak classification defined one to three septa as few denoting category II lesions, with four or more septa labelled as many denoting category IIF onwards lesions [13, 14]. Multiple three- to four-septa within a lesion form multiple internal compartments, making the lesion 'multiloculated’ [1]. Multilocular cystic nephroma and multilocular RCC are named examples, with the latter being sinister. Differentiation between malignant and benign entities may be difficult at times. Polycystic kidney disease can mimic a 'multiloculated' renal lesion. Thin slices will demonstrate a lack of capsule and normal intervening parenchyma between cysts in a case of cystic renal disease [2,9,18,22]. Other potential pitfalls include compressed intervening normal parenchyma interposed between cysts, fibrotic tissue, or embryonic remnants encountered in cystic Wilm's tumour [25].

A CEUSG is superior to CECT in categorizing septate lesions due to USG's higher spatial resolution and improved characterization of intralesional septa and solid components [14]. Park et al. evaluated renal lesions with both CECT and CEUSG. A CEUSG detected the thickness and morphology of septa and solid components better. This results in lesions being reclassified into a higher category, leading to increased surgical resection [3,26]. Quaia et al. devised a classification system for complex renal lesions based on enhancement characteristics of the peripheral wall, septa, and nodules. The study concluded that CEUSG had better diagnostic accuracy than CECT in diagnosing complex renal lesions [4].

Solid Component: Benign vs. Malignant

A solid component within a lesion virtually excludes the benign nature of the lesion and designates the lesion as category III or IV [9]. Detection of a solid component within a renal lesion requires contrast administration to differentiate it from uniformly hyperdense or hyperintense lesions. Subtraction of pre-contrast images from post-contrast images helps detect true enhancement on MRI [1].

The solid component morphology can vary from septal thickening to frank wall nodularity. Silverman et al. defined that any lesion bearing <25% solid component is deemed a cystic mass. They also defined category IV nodule morphology as any convex lesion >4 mm, a convex lesion bearing obtuse margins, or any size lesion with acute margins [14]. The proportion of solid components in a lesion is the second most important predictor of behaviour. The larger the solid component, the lower the overall and cancer-free survival [11]. These criteria apply to both CT and MRI.

Intralesional debris can mimic solid components on CT and MRI. Debris is a result of repeated haemorrhage or infection and is often not adhered to the lesion walls. While a solid neoplastic component adheres to the lesion wall, USG can demonstrate the mobility of intralesional debris upon a change in patient decubitus. Multiplanar CT and MR images can confidently identify the presence of normal parenchyma around or between the lesions [1,2]. A small malignant lesion interspersed between clusters of cysts can be detected on CECT but can be missed on USG. Smaller lesions may not show colour flow on Doppler imaging [9,25]. Similarly, an iso- to hypoechoic RCC, especially with endophytic morphology, can be challenging to detect on USG [27].

Enhancement: Methods and Pitfalls

Enhancement is the most crucial feature in categorizing a renal lesion, with lesions in categories I, II, and IIF showing no enhancement and III and IV showing measurable enhancement [13]. An updated version of the Bosniak classification proposed that wall or septa enhancement must be present to classify a lesion into a category higher than IIF [14]. Vascular lesions like renal artery aneurysms and arterio-venous fistulae show intense enhancement paralleling renal vasculature [6].

Objective and precise assessment of enhancement requires drawing a region of interest (ROI) over an NCCT and CECT image at the exact location. Both ROIs should be identical and drawn over at least half of the cross-section area of the lesion [2,7]. An internal reference, like an adjoining simple cyst, or an external reference, like a gall bladder, can help make ROIs at the same location. Neovascularization is the pathological basis of lesion enhancement. Technical factors like injected contrast media and acquisition parameters also affect the assessment [9].

The papillary variant of RCC shows a slower contrast enhancement, emphasizing the importance of delayed phase acquisition. Enhancement ratios of a tumour-to-aorta in the vascular phase, tumour-to-kidney in both the vascular and nephrographic phases, and tumour heterogeneity in the vascular phase were significant and correlated inversely to the probability of harbouring a papillary subtype. The tumour-to-aorta enhancement ratio and tumour heterogeneity in the nephrographic phase were not significantly predictive of the papillary subtype [28, 29]. The clear cell variant shows maximum enhancement in the corticomedullary phase; ADC values were also found to be lower in the papillary variant among subtypes of RCC [25].

A pseudo-enhancement artifact is seen on CECT acquisition due to the volume-averaging effect between two adjacent slices. The lesion can show pseudo-enhancement due to adjoining enhancing parenchyma and calyceal contrast media [1, 2]. Lesions with hyperdense contents can be evaluated on dual-energy CT. Dual-energy CT uses an X-ray spectrum of two different kilovolts (KVs) to determine the material composition within a lesion. Post-contrast iodine overlay images can be compared with NCCT images to detect true enhancement from 'pseudo-enhancement' [11, 18, 23, 29].

Lesions with internal haemorrhage and proteinaceous contents appear hyperintense on T1 and show 'pseudo-enhancement'. Non-contrast T1 images are subtracted from post-contrast T1 images to identify even more subtle areas of true enhancement. Magnetic resonance is beneficial for evaluating hypovascular and densely calcified lesions [6, 7, 14]. Dynamic-enhanced MRI can analyze the perfusion characteristics of lesions. The study by Ho et al. postulated that if scanning is performed at a post-contrast delay of two to four minutes, setting a threshold enhancement of 15%-20% and a mean enhancement change of 100% helps in differentiation between the cyst and solid tumour with 100% sensitivity and >94% specificity. They recommended acquiring multiple, at least three, post-contrast sequences with the same scanning parameters for measurements to ascertain maximum lesion enhancement. However, this technique was found to be of limited use in lesions <1cm in size.

Risk of Malignancy

After assigning a category to a lesion, it is imperative to communicate comprehensively, including its nature whether benign or suspicious for malignancy, with a probability of the latter. The report should include a follow-up recommendation, including a timeframe to stop imaging once the benign nature of the lesion is established. The risk of malignant transformation is 5% in category IIF lesions and 50% in category III lesions [1]. The risk of malignancy in category IIF lesions increases in the presence of coexisting category III or IV lesions and with a previous history of renal carcinoma in the patient [2]. Similarly, category IIF lesions can progress to category III or IV during follow-up, with a high prevalence of malignancy [14]. Category IV lesions are almost always malignant.

Special considerations

Small Lesions

Precise lesion characterization and enhancement assessment are challenging in small lesions [1]. A renal lesion smaller than 5 mm in its greatest dimension is difficult to assess because of volume averaging with adjacent normal parenchyma and physiological contrast enhancement [2, 29]. Renal lesions smaller than 1.5 cm suffer from ‘pseudo enhancement.' They are assumed benign if they show low attenuation and circumscribed margins [14]. Lesions measuring <1 cm should be followed up annually until they attain a 1 cm dimension. A biopsy or prompt surgical excision should be performed if the lesion grows >4 cm [23]. A biopsy can stratify patients considered for surveillance, especially in patients with RCC, after deciding subtype [11].

Paradoxically, even smaller lesions in young patients with the syndromic association are labelled as the intermediate category due to the high risk of malignancy. A CEMRI and diffusion-weighted imaging (DWI) can confirm the presence of solid components in such cases [2]. Clear-cell RCC of even small size may show necrosis and heterogeneity on CECT and CEMRI. Corticomedullary-phase CEMR images are instrumental in the demonstration of smaller malignant lesions. A study by Kim et al. showed higher CT and MRI diagnostic accuracy in detecting smaller lesions. An MRI has higher sensitivity than a CT. However, specificity remained low for both modalities [23, 29, 30].

A small angiomyolipoma (AML) can be differentiated from RCC based on fat attenuation in the lesion on NCCT. On MRI, AML shows an angular interface with renal parenchyma and a drop in signal intensity of >25% in opposed phase images, as quoted by Woo et al. Woo et al. also suggested that oncocytoma can be differentiated from RCC on CT with 100% specificity. The findings favouring an oncocytoma are as follows: nephrographic phase enhancement >32 HU, arterial phase enhancement >500%, and venous phase washout >50% [28].

Lesions With Indeterminate Imaging Features

A renal lesion with NCCT attenuation of 20 to 50 HU or a postcontrast enhancement of 10-15 HU falls in the grey area to establish its enhancing nature. It might be due to pseudo-enhancement, as seen in small cortical cysts, or a small neoplasm within a cyst [29]. Bertolotto et al. encountered a few such papillary tumours in their study [31]. The above study also cited the advantage of CEUSG in detecting intralesional vascularity, aiding in differentiating hypovascular papillary tumours from other cystic lesions. Pitfalls in evaluating enhancement can be avoided by using uniformly thin and overlapping slices, matching the location and area of ROI placed within the lesion [1]. Subtraction MR imaging can be a problem-solving tool for characterizing Bosniak IIF and III cysts, which show no appreciable enhancement on CT.

Fat-Containing Lesions

Demonstration of macroscopic fat on NCCT in a solid renal lesion aids in diagnosing AML [26]. However, a fat-containing lesion with coarse calcifications suggests RCC rather than AML. Infiltration of renal sinus fat by an RCC shows spurious fat attenuation areas in a lesion and can mimic an AML. An AML may contain microscopic fat, which can be detected by chemical shift MRI or the Dixon technique with a fat-only image dataset [26,32]. Such cases should undergo surgical resection to differentiate between RCC, lipid-poor AML, and oncocytoma. Hyperdensity on a nonenhanced scan and an arterial/delayed enhancement ratio of >1.5 favour poor lipid AML. An AML of size >4 cm and those having intralesional arterial aneurysms of size 5mm or more have a high risk of spontaneous internal haemorrhage. Such lesions are amenable to endovascular embolization [23]. The presence of a central scar, high ADC values, and segmental enhancement pattern favour oncocytoma over RCC [26, 33]. Angiomyolipoma with epithelial cysts (AMLEC) is a recently described lipid-poor AML variant that resembles other renal lesions on imaging [34].

Infiltrative Lesions

Lesions with infiltrative growth patterns cause kidney enlargement with diffuse involvement without distortion of the reniform shape. They lack capsules and show indistinct margins. The involved kidneys show large areas of hypoenhancement [6,26]. Transitional cell carcinoma (TCC), RCC, lymphoma, leukaemia, and metastasis are lesions with infiltrative growth patterns. Benign conditions like pyelonephritis and AML with haemorrhage mimic infiltrative lesions [29]. Bilateral infiltrative lesions with noncalcified retroperitoneal lymphadenopathy and a history of lymphoma, especially non-Hodgkin's lymphoma, favour a diagnosis of renal lymphoma [2, 29]. Otherwise, the mass should be biopsied for a definite diagnosis [6]. Transitional cell carcinoma of the renal collecting system and RCC should also be differentiated, as surgical management differs. Transitional cell carcinoma is frequently multifocal with preserved renal contours [34]. Epithelial and mesenchymal benign tumours are rare and show heterogeneous solid and cystic compositions and an infrequent exophytic appearance. These are mistaken for malignant lesions and classified as Bosniak category IV lesions due to their sizeable stromal solid component [2,18].

Renal parenchymal metastases are usually multiple and bilateral in distribution. The presence of an infiltrating renal lesion in a patient with a diagnosed primary malignancy should raise suspicion of renal metastases. Renal metastases are commonly seen with lung, breast, and bowel primaries. Differentiation from a Bosniak IV lesion is difficult. Multiplicity, bilaterality, and small uniform size favour metastasis [18]. It is worth mentioning that papillary RCC also demonstrates bilaterality and multiplicity with synchronous or metachronous multiple lesions [29].

Tumour Mimics

Pseudo-lesions are normal anatomical variants or conditions mimicking a lesion. Anatomical variants such as a dromedary hump, a hypertrophied column of Bertin, and persistent fetal lobulations of the kidney can mimic a mass. Tumor mimics show enhancement and corticomedullary differentiation similar to normal parenchyma in all phases [6]. Post-infective cortical scars can distort medullary pyramids by clubbing calyces [32]. 

Inflammatory pathologies like pyelonephritis and abscesses can mimic cystic and necrotic renal neoplasms [6]. A renal abscess can be a complication of an ascending urinary tract infection or a hematogenous spread from another source. Perinephric inflammatory fat stranding, air foci within the lesion, and central diffusion restriction on MRI favor the diagnosis of an abscess [18]. A CEUSG shows a peripheral rim of increased vascularity in such cases. USG-guided diagnostic or therapeutic aspiration of pus can be done [4]. Xanthogranulomatous pyelonephritis is a chronic infective pathology seen in patients with uncontrolled diabetes and obstructive renal calculi. Renal involvement is more commonly diffuse, with pathognomonic 'bear paw appearance' comprising wedge-shaped hypoenhancing areas radiating from the papilla to the periphery and rim-enhancing collections [35].

Polycystic Kidney Disease

Autosomal dominant polycystic kidney disease (ADPKD) is the most common in this group. Other entities include dialysis-induced cystic kidney disease, senile renal cysts, and lithium toxicity [18]. Lithium toxicity-induced cysts are 1-2 mm in size and are seen in cortical and medullary locations [36]. The risk of malignant transformation is comparable to that of the general population, except for dialysis patients. The hallmark of ADPKD is the strong familial association and extrarenal involvement, comprising cysts in the liver, pancreas, and intracranial ‘berry’ aneurysms. Senile renal cysts are also associated with hepatic cysts but lack a hereditary pattern [10]. A distinct subtype is localized cystic disease of the kidney, with the cysts localized to one portion of a single kidney. Renal function remains normal in such cases, a differentiating feature from multicystic dysplastic kidney disease seen in an adult population [25]. Glomerulocystic kidney disease presents as cortical, subcortical, and subcapsular cysts [36]. Cystic RCC, or RCC with large cystic components, is uncommon. It is seen in younger age groups and shows less aggressive behaviour with a better prognosis [18,34,37].

Limitations of Bosniak classification

The non-predictive categorization of malignancy due to the lesion's size and interval of growth and the inability to differentiate cystic from necrotic components are known limitations of this classification. The presence of an enhancing solid component was the strongest predictor of the lesion's malignant or aggressive nature [14]. The Bosniak classification has yet to incorporate newer imaging modalities and techniques, such as iodine mapping of lesions on CECT and functional MRI models like diffusion, perfusion chemical shift imaging, and CEUSG [14, 25]. Technical and patient-related factors responsible for altered enhancement are also not accounted for [29].

## Conclusions

The Bosniak classification is the most widely used system for classifying renal lesions based on morphological and structural features. The aim is to improve overall specificity, use uniform terminology, assign a category to a lesion, and reduce unnecessary interventions. Despite technical advances and scientific knowledge gained over the last several decades, the Bosniak classification still needs to be explored further. The roles of CEUSG and CEMRI need to be tested further before wider clinical implementation. The authors hope that this scoping review of renal lesions with an emphasis on cross-sectional imaging will help radiologists and clinicians in patient management. The authors have discussed imaging nuances to enhance the clarity and specificity of the Bosniak classification. This review will be useful in practice, serve as a base for further study, and help in future significant updates. Renal lesions are commonly encountered yet are challenging to characterize. Complex renal lesions must be characterized precisely and communicated clearly to the concerned clinician for appropriate management.
